# Iowa’s Veterinary Law

**Published:** 1900-04

**Authors:** 


### LEGISLATION.

### IOWA'S VETERINARY LAW.

*An Act to Regulate the Practice of Veterinary Medicine, Surgery, and Dentistry in the State of Iowa, and providing penalties for the violation thereof.*

Be it enacted by the General Assembly of the State of Iowa:

SECTION 1. That it shall be unlawful for any person to practice veterinary medicine, surgery, or dentistry in this State who shall not have complied with the provisions of this act.

SEC. 2. Any person who has practised the profession of veterinary medicine, surgery, or dentistry in this State for a period of five years immediately preceding the passage of this act may be deemed eligible to registration as an existing practitioner, and receive a certificate of registration, upon presentation to the Secretary of the Board of Veterinary Medical Examiners, which shall be hereinafter constituted, his sworn affidavit and letters of recommendation from ten reputable freeholders and stock owners in his locality, all such applications to be made on or before January 1, 1901.

SEC. 3. Any person who is a graduate of a legally chartered and authorized veterinary college or veterinary department of any university or agricultural college, at the time of the passage of this act, or who shall hold a diploma from such institutions prior to 1901, shall be entitled to registration as an existing practitioner upon the presentation of his diploma, duly verified.

SEC. 4. The Governor of the State shall appoint a Board of Examiners, within sixty days after the passage of this act, said Board to be known as the State Board of Veterinary Medical Examiners. This Board shall consist of three qualified veterinarians, residents of the State, each of whom shall be a graduate of a legally chartered and authorized veterinary college or veterinary department of any university or agricultural college, and who shall be of good standing in the profession.

One of these members shall be appointed for one year; one for two years, and each succeeding appointment shall be for three years.

Each shall hold office until his successor is duly appointed and qualified.

No member of any veterinary college or veterinary department of the State university or agricultural college, or any person connected therewith, shall be eligible to appointment upon said Board.

The Governor shall fill any vacancy which shall occur on the Board, and may remove any member of said Board for continued neglect of duty, for incompetency, unprofessional or dishonorable conduct.

SEC. 5. This Board shall have power to make all needed regulations for its government and proper discharge of its duties in accordance with this act, and shall have power to administer oaths and take testimony concerning all matters within its jurisdiction.

SEC. 6. The meetings of the Examining Board shall be held at least once a year, or at such times and places as it may elect. At any meeting of the Board a majority shall constitute a quorum to transact business or to conduct examinations.

SEC. 7. Said Board shall receive applications for registration, according to Sections 2 and 3 of this act, and shall issue a certificate of qualification to all applicants who conform to the requirements for such registration, signed by the members of the Board, provided that the certificate thus granted specifically and plainly states whether or not the one to whom it is granted is a graduate or non-graduate in veterinary medicine. Such certificate shall be conclusive as to the rights of the lawful holder of the same to practice veterinary medicine, surgery, or dentistry in this State.

SEC. 8. The fee for registration shall be five dollars ($5), payable in advance to the Secretary of the Board.

SEC. 9. From and after January 1, 1901, any person not heretofore authorized and eligible to practice veterinary medicine, surgery, and dentistry in this State under Section 2 hereof, and desiring to enter upon such practice, shall be a graduate of a legally chartered and recognized veterinary college or veterinary department of any university or agricultural college, and shall pass the examination required by said State Board of Veterinary Medical Examiners.

The fee for such examination shall be fifteen dollars ($15), payable in advance to the Secretary of the Board. The applicant shall be at least twenty-one years of age and of good moral character. Any person conforming to these requirements shall receive a license to practice veterinary medicine, surgery, or dentistry within this State, signed by members of the Board, which license shall be recorded in the office of the recorder of the county in which said person resides, the recording fee to be paid by the holder of certificate.

SEC. 10. The Board shall keep a register of all registered practitioners in the State, setting forth such facts as the Board shall see fit. All fees accruing under this act shall be held by the Treasurer of the Board, who shall execute a good and sufficient bond to said Board to faithfully discharge his duties, and who shall pay out such funds only on vouchers certified by a majority of said Board.

SEC. 11. Each member of said Board shall be entitled to receive five dollars ($5) per diem, also actual and necessary travelling expenses incurred while actually engaged in the discharge of his official duties, provided such compensation and expenses do not exceed said income of fees accruing under this act.

SEC. 12. Any person violating any of the provisions of this act shall be guilty of a misdemeanor, and upon conviction shall be punished by a fine of not less than twenty-five dollars nor more than one hundred dollars, or by imprisonment in the county jail for a period of not more than thirty days for each and every such offence.

It shall be the duty of the county attorney of the county in which violation occurs to conduct all proceedings against violators of this act.

SEC. 13. Nothing in this act shall be construed to apply to commissioned veterinarians in the United States army or to persons who dehorn cattle, or castrate domestic animals, or to persons who gratuitously treat diseased animals.

SEC. 14. Any person who shall, without having been authorized so to do, legally append any veterinary title to his name, or shall assume or advertise any veterinary title in such manner as to convey the impression that he is a lawful practitioner of veterinary medicine, or any of its branches, shall be guilty of a misdemeanor and punished according to the provisions of Section 12 of this act.

SEC. 15. In case the examination of any person shall prove defective and his name be not registered, he shall be permitted to present himself for re-examination within any period not exceeding twelve months next thereafter, and no charges shall be made for re-examination.

SEC. 16. The Board shall render under oath annually, on January 1st, to the Executive Council an account of all fees collected, per diem, and expenses paid, and pay over the balance into the State treasury.

---

Dr. W. B. E. Miller, recently in charge of the Quarantine Station at Garfield, New Jersey, has been succeeded at that point by G. W. Pope, of Boston, Mass. Dr. Miller will enter private practice again.

